# Aging exacerbates hypertension-induced cerebral microhemorrhages in mice: role of resveratrol treatment in vasoprotection

**DOI:** 10.1111/acel.12315

**Published:** 2015-02-09

**Authors:** Peter Toth, Stefano Tarantini, Zsolt Springo, Zsuzsanna Tucsek, Tripti Gautam, Cory B Giles, Jonathan D Wren, Akos Koller, William E Sonntag, Anna Csiszar, Zoltan Ungvari

**Affiliations:** 1 Reynolds Oklahoma Center on Aging, Department of Geriatric Medicine, University of Oklahoma Health Sciences Center975 NE 10th Street, Oklahoma City, OK, 73104, USA; 2 Department of Pathophysiology and Gerontology and Szentagothai Research Center, University of PecsSzigeti Street 12, 7624, Pecs, Hungary; 3 Department of Physiology, University of Oklahoma Health Sciences Center975 NE 10th Street, Oklahoma City, OK, 73104, USA; 4 Oklahoma Medical Research Foundation, Arthritis & Clinical Immunology Research Program825 Northeast 13th Street, Oklahoma City, OK, USA; 5 Department of Biochemistry and Molecular Biology, University of Oklahoma Health Sciences Center975 NE 10th Street, Oklahoma City, OK, 73104, USA; 6 The Peggy and Charles Stephenson Cancer Center, University of Oklahoma Health Sciences Center975 NE 10th Street, Oklahoma City, OK, 73104, USA

**Keywords:** arteriole, dementia, microbleed, NADPH oxidase, oxidative stress, cognitive impairment

## Abstract

Recent studies demonstrate that aging exacerbates hypertension-induced cognitive decline, but the specific age-related mechanisms remain elusive. Cerebral microhemorrhages (CMHs) are associated with rupture of small intracerebral vessels and are thought to progressively impair neuronal function. To determine whether aging exacerbates hypertension-induced CMHs young (3 months) and aged (24 months) mice were treated with angiotensin II plus L-NAME. We found that the same level of hypertension leads to significantly earlier onset and increased incidence of CMHs in aged mice than in young mice, as shown by neurological examination, gait analysis, and histological assessment of CMHs in serial brain sections. Hypertension-induced cerebrovascular oxidative stress and redox-sensitive activation of matrix metalloproteinases (MMPs) were increased in aging. Treatment of aged mice with resveratrol significantly attenuated hypertension-induced oxidative stress, inhibited vascular MMP activation, significantly delayed the onset, and reduced the incidence of CMHs. Collectively, aging promotes CMHs in mice likely by exacerbating hypertension-induced oxidative stress and MMP activation. Therapeutic strategies that reduce microvascular oxidative stress and MMP activation may be useful for the prevention of CMHs, protecting neurocognitive function in high-risk elderly patients.

## Introduction

Hypertension is one of the most prevalent diseases of aging. ∼80% of elderly patients (≥65 years of age) have systolic hypertension, which has deleterious effects on the cerebral circulation and the brain. There is strong clinical evidence that in the elderly hypertension leads to neural injury and impairs neuronal function, promoting the development of vascular cognitive impairment (VCI) (Gorelick *et al*., 2011) and gait dysfunction (de Laat *et al*., 2011). Experimental studies extend the clinical findings demonstrating that similar levels of hypertension result in more significant cognitive decline in aged mice, as compared to young mice (Csiszar *et al*., 2013; Toth *et al*., 2013b). The negative impact of hypertension on CNS function is due to pathological alterations of both the large cerebral arteries and the cerebral microvessels. While the deleterious effects of hypertension of large vessel atherosclerosis and development of debilitating strokes are well documented (Rajan *et al*., 2014), the microvascular mechanisms by which aging exacerbates hypertension-induced neuronal dysfunction are less understood.

Recent studies suggest that in addition to causing blood–brain barrier disruption, microvascular rarefaction and neurovascular uncoupling (Kazama *et al*., 2004; Girouard *et al*., 2007; Toth *et al*., 2013b) hypertension also promotes cerebral microhemorrhages (Wakisaka *et al*., 2010a,b) (CMHs; <5 mm in diameter). Cerebral microhemorrhages, also known as cerebral microbleeds, are associated with rupture of small intracerebral vessels and are thought to progressively impair neuronal function (Poels *et al*., 2012; Lei *et al*., 2013). Recent evidence suggests that CMHs also contribute to the pathogenesis of Alzheimer's disease (AD) (Yates *et al*., 2011) and may explain the increased risk and severity of AD in hypertension (Guo *et al*., 2001). Moreover, the presence of CMHs is an independent risk factor for subsequent large-sized hemorrhages (Bokura *et al*., 2011). The pathogenesis of CMHs was shown to involve weakening of the vessel wall by oxidative stress-dependent activation of matrix metalloproteinases (MMPs) (Wakisaka *et al*., 2010a,b). Despite the clinical importance of CMHs (Fisher *et al*., 2010), the causal link among aging, hypertension, and CMH incidence and age-related changes in redox-regulated vascular MMP activation are not well understood and there are no effective treatments available for prevention.

This study was designed to test the hypothesis that aging promotes the development of CMHs by exacerbating hypertension-induced oxidative stress and redox-sensitive activation of MMPs in the cerebrovasculature. A prediction based on this hypothesis is that pharmacological treatments that attenuate vascular oxidative stress should prevent/delay development of CMHs in aging. To test our hypotheses, we induced hypertension in young and aged mice (by treatment with angiotensin II (Ang II)) and L-NAME (Nω-nitro-l-arginine methyl ester hydrochloride, inhibitor of nitric oxide synthase) and compared the incidence, size, and localization of CMHs. To elucidate the mechanisms contributing to age-related changes in CMH incidence, hypertension-induced vascular ROS production and MMP activation were assessed. We also tested the protective effects of treatment with resveratrol, which is known to significantly attenuate vascular oxidative stress in aging (Pearson *et al*., 2008; Ungvari *et al*., 2009, 2010; Csiszar *et al*., 2012).

## Results

### Aging exacerbates hypertension-induced spontaneous CMHs in mice: prevention by resveratrol treatment

**Fig 1:**
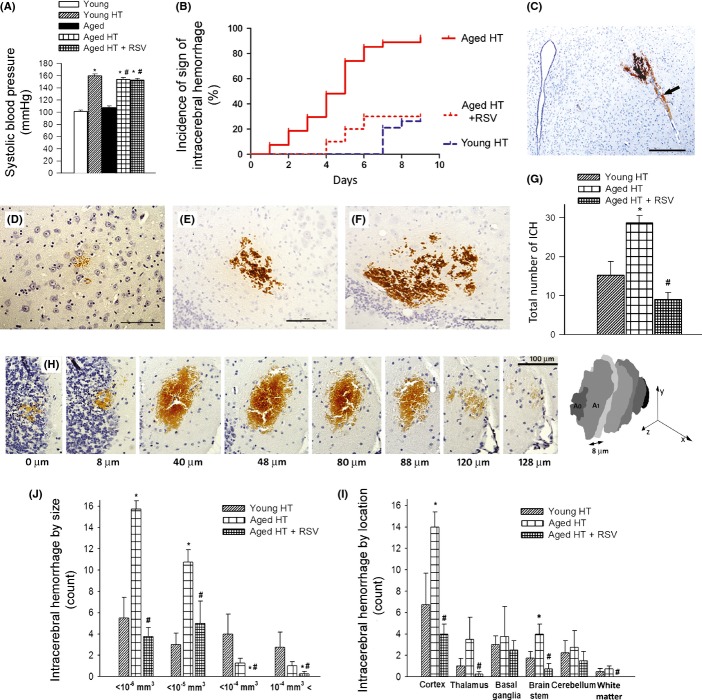
Aging exacerbates hypertension (HT)-induced spontaneous cerebral microhemorrhages (CMHs) in mice: prevention by resveratrol treatment. Shown are (A) the effect of treatment with angiotensin II plus L-NAME (HT groups) on systolic blood pressure and (B) the incidence of neurological signs of intracerebral hemorrhage in young (3 months, *n* = 20), aged (24 months, *n* = 20), and resveratrol-treated aged (*n* = 20) C57BL/6 mice. Data are mean ± SEM (*n* = 20 for each group). *P < 0.05 vs. Young, ^#^*P* < 0.05 vs. Aged. (C): Representative image of a CMH stained by diaminobenzidine (scale bar = 200 μm). Black arrow points to a cerebral intraparenchymal arteriole in close proximity to the hemorrhage. (D–F): Representative images of patchy CMHs of different sizes (scale bar = 100 μm). (G): Total number of CMHs throughout the entire brain of young, aged, and resveratrol-treated aged mice. Data are mean ± SEM. *P < 0.05 vs. Young, ^#^*P* < 0.05 vs. Aged (*n* = 20 in each group). Panel H illustrates the reconstruction of the volume of a confluent CMH (see Methods). (I): The distribution of CMHs by size and by location (J) in each experimental group. Note that aging increases the number of small CMHs predominantly in the cortex and brain stem, whereas resveratrol treatment in aged mice significantly reduces CMH incidence. Data are mean ± SEM. *P < 0.05 vs. Young, ^#^*P* < 0.05 vs. Aged (*n* = 20 in each group).

Blood pressure was significantly increased in both young and aged mice treated with Ang II plus L-NAME, as shown in Fig.1A. Resveratrol treatment did not significantly affect blood pressure (Fig.1A). We found that during the experimental period, 26% of hypertensive young mice showed clinically manifest signs of intracerebral hemorrhage (as assessed by neurological examination). In contrast, 93% of hypertensive aged mice developed signs of intracerebral hemorrhage during the experimental period, which manifested significantly earlier than those in young mice (average time to occurrence: 4.0 ± 0.3 and 7.2 ± 0.2 days postinduction of hypertension; *P* < 0.01; Fig.1B). Resveratrol treatment significantly decreased the incidence of clinically manifest intracerebral hemorrhages in aged hypertensive mice (to 30%) and delayed their occurrence (average time to occurrence: 5 ± 0.5 days postinduction of hypertension; *P* < 0.01 vs. untreated). The difference between the cumulative incidence curves is statistically highly significant (*P* < 0.0001 hypertensive aged vs. hypertensive young mice, *P* < 0.001 hypertensive aged vs. resveratrol-treated hypertensive aged mice; Mantel–Cox log-rank test).

Histological analysis confirmed that all mice with signs of intracerebral hemorrhage developed multiple CMHs that were distributed widely in the brain (Fig.1J) and varied in appearance (representative images of confluent and patchy CMHs are shown in Fig.1D–F). When the cerebral vessels associated with the CMHs were clearly distinguishable, their internal diameter was found to be in the range of ∼10–20 μm (Fig.1C). No normotensive mice developed neurological signs of hemorrhages or histologically detectable CMHs (data not shown). In hypertensive young mice showing signs of intracerebral hemorrhage, only a few CMHs were found, whereas the same level of hypertension in aged mice resulted in a significantly increased number of CMHs (Fig.1G). Resveratrol treatment not only delayed the occurrence of signs of intracerebral hemorrhage but also significantly reduced the number of CMHs in the brain of hypertensive aged mice (Fig.1B,G). The volume distribution of CMHs is shown in Fig.1I. Aging predominantly increased smaller CMHs. As shown in Fig.1J, in hypertensive aged mice, there were more CMHs predominantly in cerebral cortex and brainstem than in hypertensive young mice. The number of CMHs in the thalamus also tended to increase in hypertensive aged mice, although the difference did not reach statistical significance. Resveratrol treatment in hypertensive aged mice significantly reduced the number of CMHs in cerebral cortex, thalamus, and brainstem (Fig.1J).

### Increased incidence of CMHs is associated with early gait dysfunction in hypertensive aged mice: prevention by resveratrol treatment

**Fig 2:**
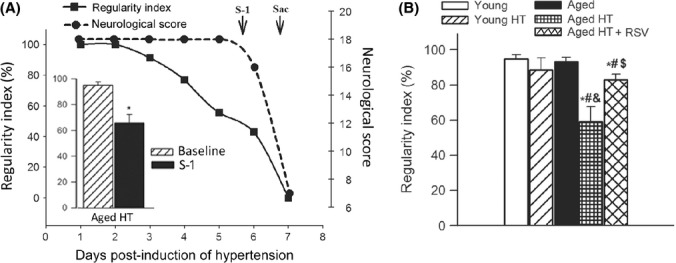
Increased incidence of cerebral microhemorrhages (CMHs) is associated with early gait dysfunction in hypertensive aged mice: prevention by resveratrol treatment. Panel A shows that dysfunction of gait coordination (indicated by changes in the regulatory index) is manifested before the onset of signs of intracerebral hemorrhage detectable by standard neurological examination. Regularity index is a measure of interpaw coordination. In healthy, fully coordinated animals, its value is 100%. Inset: significant decline in the regularity index between the day before induction of hypertension and the day before the onset of clinically detectable hemorrhage (S-1) in hypertensive aged mice. Data are mean ± SEM. *P < 0.05 vs. Day baseline. (*n* = 20). B: Data from cross-sectional studies. Shown are changes in regularity index induced by treatment with angiotensin II plus L-NAME (HT groups) in young (3 months, *n* = 20), aged (24 months, *n* = 20), and resveratrol-treated aged (*n* = 20) C57BL/6 mice on day 5 postinduction of hypertension. Note that aging exacerbates hypertension-induced decline in the regulatory, which is reversed by resveratrol treatment. Data are mean ± SEM. *P < 0.05 vs. Young, ^#^*P* < 0.05 vs. aged, ^&^*P* < 0.05 vs. Young HT, ^$^*P* < 0.05 vs. Aged HT (*n* = 20 in each group).

Figure2A shows that early and progressive dysfunction of gait coordination in hypertensive aged mice can be detected before the onset of signs of intracerebral hemorrhage detectable by standardized neurological examination. Decline in the regularity index between the day before induction of hypertension and the day before the onset of clinically detectable hemorrhage in hypertensive aged mice was significant (Fig.2A, inset), validating the concept that description of motor function status (including deficit in interlimb coordination and temporal asymmetry) by computerized gait analysis can predict the occurrence of CMHs. For cross-sectional studies, a cohort of animals, which had not yet shown clinical signs of hemorrhage, was sacrificed on day 5 postinduction of hypertension. We found that the regularity index was similar in normotensive young and aged mice, whereas aging exacerbated hypertension-induced decline in gait function (Fig.2).

### Aging exacerbates hypertension-induced vascular oxidative stress: prevention by resveratrol treatment

**Fig 3:**
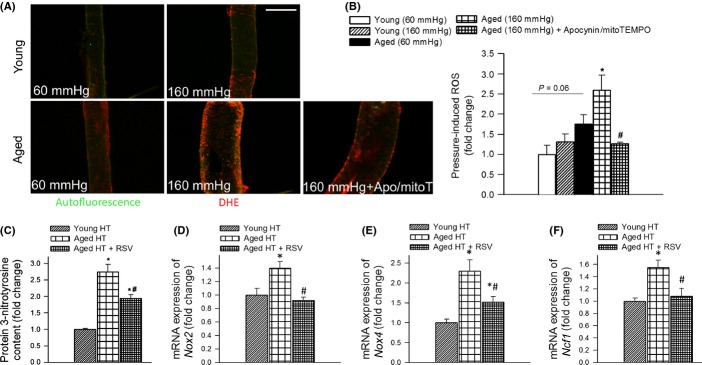
Aging exacerbates hypertension-induced vascular oxidative stress. A: Representative confocal images showing stronger dihydroethidium (DHE) staining (red fluorescence) indicating increased O_2_^.−^ production in high pressure-exposed MCAs isolated from aged mice as compared to MCAs isolated from young mice. MCAs were pressurized at 60 and 160 mmHg for 6 h. Note that high pressure-induced oxidative stress in aged MCAs was significantly attenuated by the NADPH oxidase inhibitor apocynin and the mitochondria-targeted antioxidant mitoTEMPO. Green autofluorescence is shown for orientation purposes (original magnification: 20×, scale bar: 100 μm). Bar graphs (B) are summary data. Data are means ± SEM (*n* = 6 in each group). *P < 0.05. vs. Young (160 mmHg),^#^*P* < 0.05 vs. Aged (160 mmHg). C: Cortical 3-nitrotyrosine content in hypertensive (HT) young, aged, and resveratrol-treated aged mice (*n* = 6 in each group). Data are mean ± SEM. *P < 0.05 vs. Young HT, ^#^*P* < 0.05 vs. Aged HT. D, E, and F show hypertension-induced mRNA expression of NADPH oxidase subunits *Nox2*, *Nox4,* and *Ncf1* in cerebral arteries of young, aged, and resveratrol-treated aged mice. Data are mean ± SEM (*n* = 6 in each group). *P < 0.05 vs. Young HT, ^#^*P* < 0.05 vs. Aged HT.

We performed an implicit relationship analysis of entities (relevant vascular pathologies, genes, etc.) within the published literature to identify potential pressure-induced factors involved in cerebrovascular fragility and development of CMHs in aging using the IRIDESCENT text mining package (Wren & Garner, 2004). This analysis confirmed the conclusions based on the results of previous experimental studies in mouse models of intracerebral hemorrhage (Wakisaka *et al*., 2010a,b), identifying oxidative stress and MMP activation as important factors potentially associated with CMHs in aging. Because previous studies showed that high intraluminal pressure, via increased wall tension-dependent cellular stretch, is the primary stimulus for increased vascular ROS production in hypertension (Ungvari *et al*., 2003), we compared pressure-induced production of ROS in cerebral arteries isolated from young and aged mice. We used the redox-sensitive dye dihydroethidium (DHE) to examine pressure-induced production of O_2_^.−^ in mouse cerebral arteries. We found that nuclear DHE fluorescence (Fig.3A,B) was significantly stronger in arteries exposed to high pressure as compared to vessels of the same animals exposed to 60 mmHg, indicating that high pressure increases ROS production in the cerebral arteries. Our recent studies demonstrate that increased ROS production within the smooth muscle cells contributes significantly to high pressure-induced vascular oxidative stress in aging (Springo *et al*., 2015). Comparison of vessels isolated from aged mice to vessels isolated from young mice showed that aging significantly increases high pressure-induced ROS production in cerebral arteries (Fig.3A,B). High pressure-induced vascular ROS production was significantly attenuated by the co-administration of the NADPH oxidase inhibitor apocynin and the mitochondrial ROS scavenger mitoTEMPO (Fig.3A,B). 3-nitrotyrosine (3-NT) content in the cerebral cortex was significantly elevated in hypertensive aged mice (Fig.3C) consistent with the exacerbation of hypertension-induced oxidative/nitrosative stress in the aged brain. Resveratrol treatment significantly reduced 3-NT content in the cortex of hypertensive aged mice (Fig.3C). These results support the concept that antioxidative effects of resveratrol play a central role in its microvascular protective effects in aging.

NADPH oxidases are important sources of ROS in the cerebral vasculature, whose increased expression and activity contribute to hypertension-induced vascular oxidative stress (Girouard *et al*., 2006) (Fig.3A,B). We found that in isolated cerebral vessels (Fig.3D–F) and cortical samples (data not shown) of hypertensive aged mice, mRNA expression of the NADPH oxidase subunits *Nox2*, *Nox4,* and *Ncf1* was upregulated compared to hypertensive young mice. Treatment of aged mice with resveratrol resulted in significant downregulation of these NADPH oxidase subunits (Fig.3D–F).

### Aging exacerbates hypertension-induced MMP activation: prevention by resveratrol treatment

**Fig 4:**
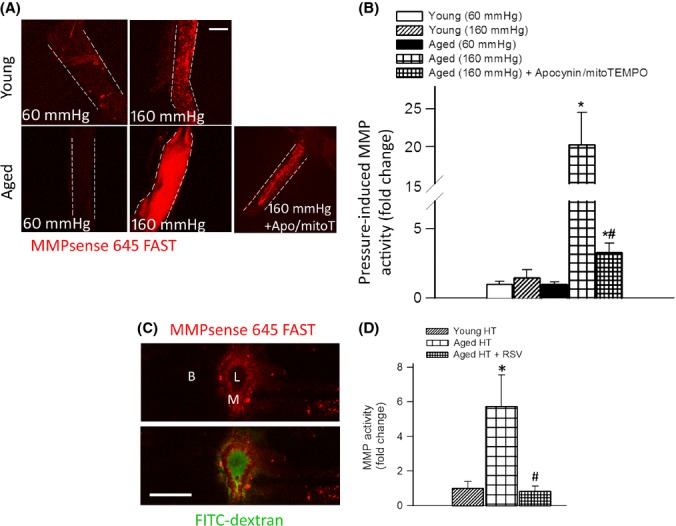
Aging exacerbates hypertension-induced redox-sensitive matrix metalloproteinases (MMP) activation. Representative compressed Z stacks of confocal images of MCAs showing stronger MMPsense 645 FAST fluorescence (red) in high pressure-exposed MCAs isolated from aged mice as compared to MCAs isolated from young mice, indicating increased MMP activation. MCAs were pressurized at 60 and 160 mmHg for 6 h. MMPsense 645 FAST becomes fluorescent upon cleavage by activated MMPs. Note that high pressure-induced MMP activation in aged MCAs was significantly attenuated by the NADPH oxidase inhibitor apocynin and the mitochondria-targeted antioxidant mitoTEMPO (original magnification: 20×, scale bar: 100 μm). Bar graphs (B) are summary data. Data are means ± SEM (*n* = 6 in each group). *P < 0.05. vs. Young (160 mmHg), ^#^*P* < 0.05 vs. Aged (160 mmHg). (C) Representative confocal image of the cross section of a cerebral intraparenchymal arteriole from an aged hypertensive mouse injected with the MMPsense 645 FAST substrate (scale bar: 50 μm). Note the strong red fluorescence in the vascular wall indicating increased MMP activity. Intraluminal FITC-dextran is shown for orientation purposes. L (lumen), M (media), B (brain parenchyma). (D): Hypertension-induced MMP activation assessed using the MMPsense 645 FAST fluorescent method, in young, aged, and resveratrol-treated aged mice (*n* = 6 in each group). Data are mean ± SEM. *P < 0.05 vs. Young HT, ^#^*P* < 0.05 vs. Aged HT.

Redox-sensitive activation of MMPs is thought to play a central role in the pathogenesis of CMHs (Wakisaka *et al*., 2010a,b). Because previous studies showed that high intraluminal pressure is the primary stimulus for upregulation of vascular MMPs (Lehoux *et al*., 2004), we compared pressure-induced MMP activation in cerebral arteries isolated from young and aged mice using the MMPsense 645 FAST substrate. We found that in young vessels, high pressure did not have any significant effect on MMP activity (Fig.4A,B). In contrast, MMP activity was substantially increased in high pressure-exposed arteries of aged mice as compared to vessels of the same animals exposed to 60 mmHg (Fig.4A,B) indicating that aging exacerbates high pressure-induced MMP activation in cerebral vessels (Fig.4B). High pressure-induced vascular MMP activation was significantly attenuated by the co-administration of the NADPH oxidase inhibitor apocynin and the mitochondrial ROS scavenger mitoTEMPO (Fig.4B).

To substantiate our findings, vascular MMP activity was also compared in hypertensive young and aged mice by *in vivo* administration of the MMPsense 645 FAST substrate. Activity of MMP-9 was barely detectable by confocal microscopy in normotensive young mice and was not increased significantly by hypertension. Representative images, shown in Fig.4C, illustrate that in hypertensive aged mice, there was strong MMP activity (indicated by the presence of the fluorescent product of the MMPsense 645 FAST substrate) localized mainly to the media of small intracerebral arteries (identified by the intraluminal FITC-dextran fluorescence). No significant MMP 645 FAST fluorescence was observed in the brain parenchyma. For quantification purposes, a plate reader-based method was used. We found that in young mice, hypertension did not have any significant effect on MMP activity (Fig.4D). In contrast, MMP activity was substantially increased in hypertensive aged mice, which was inhibited by resveratrol treatment (Fig.4D).

## Discussion

This is the first study to demonstrate that in aged mice, the same level of hypertension leads to significantly earlier onset and increased incidence of intracerebral hemorrhages, as compared to young mice (Fig.1). Importantly, aging predominantly increased the incidence of small CMHs, suggesting that aging renders small cerebral vessels significantly more vulnerable to high pressure-induced rupture. These results are in agreement with the available clinical evidence suggesting that hypertension almost exclusively increases CMHs at an old age (Romero *et al*., 2014). In humans, CMHs are an important mechanism for cognitive impairment (Seo *et al*., 2007) and their increased prevalence and incidence in aging are consistent with the documented age-related exacerbation of hypertension-induced cognitive decline in humans and laboratory animals (Csiszar *et al*., 2013; Toth *et al*., 2013b).

There are many similarities between CMHs observed in mice and humans, including the relative size of the bleedings, the clinical symptoms, and the progressive nature of the pathological process. Of note, in the mouse model, CMHs were observed frequently in the cortex (Fig.1J), whereas in humans, CMHs are often located in basal ganglia or subcortical white matter. The causes responsible for anatomic predilections for CMHs are presently not well understood and likely involve differences in the branching patterns of the cerebral arteries and pressure distribution along the microvascular network.

As small numbers of CMHs are likely difficult to detect with classical neurological examination until their aggregate volume reaches a critical level, we established a novel method to detect subtle neuronal dysfunction by analyzing gait function of mice. We found that in hypertensive aged mice, impaired gait function was manifested before other neurological assessments became positive and that it was a sensitive predictor of CMHs, which manifested significantly earlier than in young mice (Fig.2). Importantly, CMHs have been shown to be associated with gait dysfunction in humans, as well (Choi *et al*., 2012).

The age-related mechanisms responsible for increased susceptibility of the cerebral circulation to hypertension-induced rupture are likely multifaceted. Previous studies suggest a central role for oxidative stress and redox-sensitive activation of MMPs in the pathogenesis of CMHs, which are known to degrade components of the basal lamina and extracellular matrix, weakening the vascular wall (Wakisaka *et al*., 2008, 2010a). Although age-related exacerbation of hypertension-induced cerebrovascular ROS production (Fig.3) and MMP activation (Fig.4) likely importantly contributed to the increased fragility of aged cerebral arteries, further studies are evidently needed to establish a direct causal link between MMP activation and the development of CMHs in the aged brain. Increased MMP activation is likely a downstream consequence of increased hypertension-induced oxidative stress (Wakisaka *et al*., 2010b), as it can be significantly attenuated by combined inhibition of NOX oxidases and mitochondrial ROS generation (Fig4A,B). Future studies should determine whether inhibition of ROS synthesis by these sources by genetic or pharmacological means can prevent development of CMHs in aging.

Because high pressure itself appears to be the main stimulus for increased vascular ROS production (Fig.3A,B) and MMP activation (Fig.4A,B) in aging, it is likely that penetration of increased arterial pressure to the vulnerable distal portion of the cerebral microcirculation is a key factor in the development of CMHs in aging. In that regard, it is significant that hypertension in aging is associated with autoregulatory dysfunction and impaired myogenic adaptation of the cerebral resistance arteries to high pressure (Toth *et al*., 2013a,b), which likely allows sudden increases in blood pressure (e.g., during Valsalva maneuver) to cause damage to the thin-walled vessels in the brain. This effect is likely exacerbated by deficiency of NO, which amplifies the effect of angiotensin II and significantly increases the stiffness of the conduit arteries (Hu *et al*., 1997; Gao *et al*., 2014) impairing Windkessel function (normally large arteries arising from the heart function as an elastic reservoir: by distending during systole and recoiling during diastole, they dampen the fluctuation in blood pressure). This leads to increasing pulse pressure and promotes penetration of the pressure wave into the cerebral microcirculation (Tarumi *et al*., 2014). This mechanism also provides a likely explanation for the observation that aging predominantly increases the incidence of smaller CMHs.

Based on the documented association between oxidative stress and CMHs (Wakisaka *et al*., 2010b), it is predicted that interventional strategies that attenuate vascular ROS production should prevent CMHs in aged hypertensive mice. To test this hypothesis, we assessed the effect of resveratrol, which was previously shown to significantly attenuate vascular oxidative stress and MMP activity in animal models of aging (Pearson *et al*., 2008; Kaneko *et al*., 2011; Csiszar *et al*., 2012; Toth *et al*., 2014). In support of our hypothesis, we found that resveratrol treatment prevented/delayed the development of hypertension-induced CMHs in aged mice (Fig1), which was associated with preserved gait function (Fig.2). We attribute the protective effects of resveratrol to its ability to attenuate hypertension-induced oxidative stress (Fig.3) and, consequentially, MMP activation (Fig.4) in aged mice.

The cellular mechanisms by which resveratrol attenuates hypertension-induced vascular oxidative stress in aging are likely multifaceted. Hypertension in aging both upregulates NOX oxidases (Fig.3D–F) and increases mitochondrial ROS production (Dikalov & Ungvari, 2013). Thus, it is significant that resveratrol treatment results in a significant downregulation of NOX oxidases contributing to both cytoplasmic and mitochondrial oxidative stress (Nox1 and Nox4, respectively; Fig.3D,E) (Pearson *et al*., 2008; Dai *et al*., 2012; Toth *et al*., 2014). In addition, resveratrol was shown to effectively inhibit NOX activation (Zarzuelo *et al*., 2013), attenuate mitochondrial ROS production (Ungvari *et al*., 2009; Csiszar *et al*., 2012), and activate Nrf2-dependent antioxidant mechanisms (Ungvari *et al*., 2010), which may also contribute to its antioxidative effects in aged hypertensive mice. Our findings have important clinical relevance. In humans, resveratrol is well-tolerated with no reports of significant toxicity (Baur *et al*., 2012). Although the bioavailability of resveratrol in parenchymal tissues is relatively low, the vasculature, which is in direct contact with blood, is considered an ideal target for circulating resveratrol and its active metabolites (Baur *et al*., 2012). It is likely that protective effects of resveratrol against CMHs, combined with its documented efficacy to improve neurovascular coupling (Toth *et al*., 2014) and cerebromicrovascular density (Oomen *et al*., 2009), will exert beneficial neurocognitive effects in aging, especially when hypertension is also present. Further studies are warranted to test this possibility.

In conclusion, the results of this study show that aging exacerbates hypertension-induced CMHs in a mouse model that recapitulates cerebromicrovascular alterations present in elderly humans. When viewed within the broader context of prior studies, the findings presented herein suggest that aging exacerbates hypertension-induced production of ROS and activation of MMPs in the wall of cerebral vessels, which likely renders them more vulnerable to high pressure-induced rupture. Further, this is the first study to demonstrate that accumulation of CMHs in aged mice is associated with impaired gait function, which may be used to assess treatment efficiency. A third interesting aspect of this study is that resveratrol treatment confers significant protective effects against the development of CMHs in the animal model of aging used. This observation suggests the interesting possibility that it may be possible to develop therapeutic strategies that specifically disrupt pressure-induced ROS production and MMP activation for prevention of CMHs in a high-risk elderly population. As CMHs affect ∼ 36% of older individuals, future preclinical and translational studies are warranted to address this important question.

## Experimental procedures

All procedures were approved by and followed the guidelines of the Institutional Animal Care and Use Committee of OUHSC in accordance with the ARRIVE guidelines.

### Animals

Young (3 months, *n *= 40) and aged (24 months, *n *= 80) male C57BL/6 mice were purchased from the aging colony maintained by the National Institute on Aging at Charles River Laboratories (Wilmington, MA, USA). Animals were housed under specific pathogen-free barrier conditions in the Rodent Barrier Facility at the University of Oklahoma Health Sciences Center under a controlled photoperiod (12 h light; 12 h dark) with unlimited access to water.

### Induction of spontaneous intracerebral microhemorrhages and resveratrol treatment

To study spontaneous intracerebral microhemorrhages, we have used a mouse model previously characterized by the Heistad laboratory (Wakisaka *et al*., 2010a,b): mice with hypertension induced by combined treatment with angiotensin II (Ang II) and ω-nitro-l-arginine methyl ester (L-NAME). As aging is associated with increased activity of the vascular renin–angiotensin system and Ang II-dependent hypertension is common among older individuals (Wang *et al*., 2010), Ang II-dependent hypertension is a clinically highly relevant model to study age-dependent cerebrovascular alterations (Toth *et al*., 2013b). In brief, mice in each age cohort were assigned to two groups: control animals receiving vehicle and animals receiving Ang II (1000 ng min^−1^ kg^−1^ in sterile saline, via subcutaneously implanted osmotic minipumps (Toth *et al*., 2013a,b) plus the NO synthase inhibitor L-NAME (100 mg kg^−1^ day^−1^, in drinking water). Alzet mini-osmotic pumps (Durect Co, Cupertino, CA, USA) were filled either with saline vehicle or solutions of Ang II (Sigma Chemical Co., St. Louis, Missouri, USA) and were placed into the subcutaneous space of ketamine/xylazine anesthetized mice through a small incision in the back of the neck that was closed with surgical sutures. All incision sites healed rapidly without the need for any medication.

In a subgroup of aged mice, treatment with resveratrol (200 mgkg^−1^ day^−1^, p.o.) (Toth *et al*., 2014) was started 10 days prior to induction of hypertension with Ang II plus L-NAME and was continued throughout the whole experimental period. Blood pressure of the animals was recorded before the treatment and every second day during the treatment period by the tail cuff method, as described (Toth *et al*., 2013a,b). The first cohort of animals was closely monitored, and mice were sacrificed upon the occurrence of clinical signs of intracerebral hemorrhages. For cross-sectional studies, a second cohort of animals was sacrificed on day 5 postinduction of hypertension.

### Standardized neurological examination of mice

To assess the occurrence of clinically manifest intracerebral hemorrhages, neurological examination was carried out twice a day including assessment of animals' spontaneous activity, symmetry in the movement of the four limbs, forelimb outstretching, climbing ability, body proprioception, and response to vibrissae touch. Each examined animal was provided with a daily score calculated by the summation of all six individual test scores. The minimum neurological score was 3 and the maximum 18. When a consistent decline in the neurological score was observed or at day 10th of the study, mice were euthanized by CO_2_ asphyxiation.

### Analysis of gait function

Gait coordination was examined by the CatWalk System (Noldus Information Technology Inc. Leesburg, VA, USA). Using the CatWalk system, the detection of paw prints' size, pressure, and pattern during volunteer running on an illuminated glass walkway by a camera placed under the glass surface provides an automated computerized way to assess gait function and the spatial and temporal aspects of interlimb coordination (Hamers *et al*., 2006). Briefly, animals were trained to cross the walkway and then, in a dark room, had 3 consecutive runs every day in the instrument throughout the experimental period. Data were averaged across 3 runs in which the animal maintained a constant speed across the walkway. After manual identification and labeling of each footprint, the regularity index was calculated. The regularity index (%) is a fractional measure of interpaw coordination, which expresses the number of normal step sequence patterns relative to the total number of paw placements. The formula of regularity index is as follows: NSSPx4/PP × 100 (%), where NSSP represents the number of normal step sequence patterns and PP the total number of paw placements. In healthy, fully coordinated animals, its value is 100%.

### Histological analysis of intracerebral hemorrhages

Mice were euthanized and transcardially perfused with heparinized PBS for 5 min and decapitated. Subsequently, the brains were isolated and fixed in 10% formalin at room temperature for 1 day. The next day, the brains were placed in fresh 10% formalin (at 4 °C, for 2 days), then in 70% ethanol (at 4 °C, for 2 days), followed by embedding in paraffin. The brains were serially sectioned at 8 μm thickness yielding approximately 1500 sections per brain. The first two sections of every five section were stained with hematoxylin to reveal the brain structure and diaminobenzidine (DAB) to highlight the presence of hemorrhages. DAB turns into dark brown when it undergoes a reaction with peroxidases present in red blood cells therefore allowing precise detection of extravasated blood cells in the parenchyma of the brain. All stained sections were screened, and images were acquired in the evidence of a positive DAB reaction. Digital images were analyzed with imagej software (NIH) to identify the location and quantify the number and size of hemorrhages. The size of hemorrhages was estimated as follows: (area (mm^2^) of CMHs on each section) × (25 × 10^−3^ (mm): distance between successive sections).

### Implicit relationship analysis

To predict factors associated with vascular fragility and CMHs, we used the IRIDESCENT (Wren & Garner, 2004) text mining package. IRIDESCENT's 2014 database contains 615 553 recognized terms and phrases obtained from public databases (OMIM, Entrez Gene, Gene Ontology, ChemID, FDA approved drugs, and Disease Ontology terms). IRIDESCENT processed over 24 million MEDLINE records to identify co-occurrences of these terms within PubMed titles and abstracts. This creates a network of concepts, weighted by their frequency of co-occurrence. Concepts such as ‘vascular fragility’, which includes synonymous concepts such as ‘microhemorrhages’, can then be analyzed for other concepts that share a statistically significant number of co-occurring concepts in the literature. This enables related concepts to be ranked on the basis of the number and weight of the relationships they share within the literature, whether or not any published literature exists documenting a connection between the two (i.e., they are implicit rather than explicit relationships).

### Detection of high pressure-induced production of ROS in isolated cerebral arteries

Two segments of the middle cerebral arteries were isolated from the brains of young and aged mice, as reported (Toth *et al*., 2013a,b). The vessels were mounted onto two glass micropipettes in an organ chamber in oxygenated (21% O_2_, 5% CO_2_, 75% N_2_) Krebs' buffer (composed of (in mmol L^−1^): 110.0 NaCl, 5.0 KCl, 2.5 CaCl_2_, 1.0 MgSO_4_, 1.0 KH_2_PO_4_, 5.5 glucose, and 24.0 NaHCO_3_, pH ∼7.4; at 37 °C) and pressurized to 10 mmHg. Inflow and outflow pressures were controlled and measured by a pressure servo-control system (Living Systems Instrumentation, Burlington, VE, USA). The absence of leaks was verified by observing no changes in intraluminal pressure over 3 min upon turning off the pressure servo-control system. Then, vessels from the same animals were pressurized to 60 or 160 mmHg (normal pressure and high pressure group, respectively) for 4 h. To characterize high pressure-induced vascular ROS production, at the end of the incubation period, the vessels were loaded with the redox-sensitive dye DHE (Invitrogen, Carlsbad CA, USA; 3 × 10^−6^ mol L^−1^; for 30 min) as previously reported (Ungvari *et al*., 2003). After loading, the chamber was washed out five times with warm Krebs buffer, and the vessels were allowed to equilibrate for another 20 min. In additional experiments, the effect of the NADPH oxidase inhibitor apocynin (3 × 10^−6^ mol L^−1^; Cayman Chemicals, Ann Arbor, MI, USA) and the mitochondria-targeted antioxidant MitoTEMPO (10^−6^ mol L^−1^; Sigma-Aldrich, Saint Louis, MO, USA) on high pressure-induced ROS production in aged vessels was determined. After the experimental period, confocal images of the wall of the pressurized vessels were captured using a Leica SP2 confocal laser scanning microscope (Leica Microsystems GmbH, Wetzlar, Germany). Average nuclear DHE fluorescence intensities were assessed using the metamorph software (Molecular Devices LLC, Sunnyvale, CA, USA), and values for each animal in each group were averaged.

### Assessment of markers of hypertension-induced oxidative stress

As a marker of hypertension-induced oxidative/nitrosative stress, 3-NT (a marker for peroxynitrite action) was assessed in homogenates of cortical samples using the OxiSelect Protein Nitrotyrosine ELISA Kits (Cell Biolabs, San Diego, CA, USA), as reported (Toth *et al*., 2014).

### Detection of high pressure-induced activation of MMPs in isolated cerebral arteries

In separate experiments, pressure-induced MMP activity was measured in cannulated segments of the middle cerebral arteries. The arteries from young and aged animals were pressurized to 60 or 160 mmHg in the presence of MMPsense 645 FAST substrate (PerkinElmer Inc, Boston MA, USA; 3 μmol L^−1^; at 37 °C, for 6 h, in the dark). This substrate is normally optically inert. Once it is cleaved, its subunits become excitable at 649 nm and emit a red signal that can be measured as an indicator of activity of MMP 2, 3, 7, 9, 12 and 13. After the incubation period, the vessels were thoroughly rinsed, placed on a glass slide, and imaged with a Leica SP2 upright confocal microscope. In additional experiments, the effect of apocynin and mitoTEMPO on high pressure-induced MMP activation in aged vessels was determined. The detected fluorescence intensity emitted at 666 nm was measured, corrected for the background, and normalized to vessel surface area using the metamorph software (Molecular Devices LLC).

### Detection of hypertension-induced MMP activation in the cerebral vessels *in situ*

Mice from each experimental group were temporarily anesthetized with ketamine/xylazine and injected retro-orbitally with a 100 μL dose of 40 nmol L^−1^ MMPsense 645 FAST substrate. After 12 h of circulation of the substrate, animals were transcardially perfused with PBS containing 1× heparin and FITC-dextran (to highlight the vascular lumen). Then, the mice were decapitated and the brains were removed and cut in half. From the left hemisphere, the frontal cortex containing the photoactive substrate was isolated and homogenized. To quantify MMP activity, the background corrected fluorescence (Ex: 649 nm, Em: 666 nm) was measured spectrophotofluorometrically using a microplate reader and normalized to tissue weight. The right hemisphere was embedded in OCT media, and cryosectioned and confocal images of brain areas containing cross sections of penetrating small arteries were captured.

### Quantitative real-time RT–PCR

A quantitative real-time RT–PCR technique was used to analyze mRNA expression for following genes in cortical samples of mice from each experimental group: *Nox1, Nox2, Nox4, and Ncf1* (p47phox) using a Strategene MX3000 platform, as previously reported (Toth *et al*., 2014). In brief, total RNA was isolated with a Mini RNA Isolation Kit (Zymo Research, Orange, CA, USA) and was reverse transcribed using Superscript III RT (Invitrogen). Amplification efficiencies were determined using a dilution series of a standard vascular sample. Quantification was performed using the efficiency-corrected ΔΔCq method. The relative quantities of the reference genes *Hprt, Ywhaz, B2 m*, and *Actb* were determined, and a normalization factor was calculated based on the geometric mean for internal normalization. Fidelity of the PCR reaction was determined by melting temperature analysis and visualization of the product on a 2% agarose gel.

### Statistical analysis

Analysis of variance followed by Bonferroni test was used for comparison of multiple groups. Cumulative incidence of signs of hemorrhage was evaluated using a Kaplan–Meier test, and the difference among groups was analyzed by log-rank test (Mantel–Cox). A *P* value <0.05 was considered statistically significant. Data are expressed as mean ± SEM.

## References

[b1] Baur JA, Ungvari Z, Minor RK, Le Couteur DG, de Cabo R (2012). Are sirtuins viable targets for improving healthspan and lifespan?. Nat. Rev. Drug Discov.

[b2] Bokura H, Saika R, Yamaguchi T, Nagai A, Oguro H, Kobayashi S, Yamaguchi S (2011). Microbleeds are associated with subsequent hemorrhagic and ischemic stroke in healthy elderly individuals. Stroke.

[b3] Choi P, Ren M, Phan TG, Callisaya M, Ly JV, Beare R, Chong W, Srikanth V (2012). Silent infarcts and cerebral microbleeds modify the associations of white matter lesions with gait and postural stability: population-based study. Stroke.

[b4] Csiszar A, Sosnowska D, Wang M, Lakatta EG, Sonntag WE, Ungvari Z (2012). Age-associated proinflammatory secretory phenotype in vascular smooth muscle cells from the non-human primate Macaca mulatta: reversal by resveratrol treatment. J. Gerontol. A Biol. Sci. Med. Sci.

[b5] Csiszar A, Tucsek Z, Toth P, Sosnowska D, Gautam T, Koller A, Deak F, Sonntag WE, Ungvari Z (2013). Synergistic effects of hypertension and aging on cognitive function and hippocampal expression of genes involved in beta-amyloid generation and Alzheimer's disease. Am. J. Physiol. Heart Circ. Physiol.

[b6] Dai DF, Rabinovitch PS, Ungvari Z (2012). Mitochondria and cardiovascular aging. Circ. Res.

[b7] Dikalov SI, Ungvari Z (2013). Role of mitochondrial oxidative stress in hypertension. Am. J. Physiol. Heart Circ. Physiol.

[b8] Fisher M, French S, Ji P, Kim RC (2010). Cerebral microbleeds in the elderly: a pathological analysis. Stroke.

[b9] Gao YZ, Saphirstein RJ, Yamin R, Suki B, Morgan KG (2014). Aging impairs smooth muscle-mediated regulation of aortic stiffness: a defect in shock absorption function?. Am. J. Physiol. Heart Circ. Physiol.

[b10] Girouard H, Park L, Anrather J, Zhou P, Iadecola C (2006). Angiotensin II attenuates endothelium-dependent responses in the cerebral microcirculation through nox-2-derived radicals. Arterioscler. Thromb. Vasc. Biol.

[b11] Girouard H, Park L, Anrather J, Zhou P, Iadecola C (2007). Cerebrovascular nitrosative stress mediates neurovascular and endothelial dysfunction induced by angiotensin II. Arterioscler. Thromb. Vasc. Biol.

[b12] Gorelick PB, Scuteri A, Black SE, Decarli C, Greenberg SM, Iadecola C, Launer LJ, Laurent S, Lopez OL, Nyenhuis D, Petersen RC, Schneider JA, Tzourio C, Arnett DK, Bennett DA, Chui HC, Higashida RT, Lindquist R, Nilsson PM, Roman GC, Sellke FW, Seshadri S, American Heart Association Stroke Council CoE, Prevention CoCNCoCR, Intervention, Council on Cardiovascular S, Anesthesia (2011). Vascular contributions to cognitive impairment and dementia: a statement for healthcare professionals from the american heart association/american stroke association. Stroke.

[b13] Guo Z, Qiu C, Viitanen M, Fastbom J, Winblad B, Fratiglioni L (2001). Blood pressure and dementia in persons 75+ years old: 3-year follow-up results from the Kungsholmen Project. J. Alzheimers Dis.

[b14] Hamers FP, Koopmans GC, Joosten EA (2006). CatWalk-assisted gait analysis in the assessment of spinal cord injury. J. Neurotrauma.

[b15] Hu CT, Chang KC, Wu CY, Chen HI (1997). Acute effects of nitric oxide blockade with L-NAME on arterial haemodynamics in the rat. Br. J. Pharmacol.

[b16] Kaneko H, Anzai T, Morisawa M, Kohno T, Nagai T, Anzai A, Takahashi T, Shimoda M, Sasaki A, Maekawa Y, Yoshimura K, Aoki H, Tsubota K, Yoshikawa T, Okada Y, Ogawa S, Fukuda K (2011). Resveratrol prevents the development of abdominal aortic aneurysm through attenuation of inflammation, oxidative stress, and neovascularization. Atherosclerosis.

[b17] Kazama K, Anrather J, Zhou P, Girouard H, Frys K, Milner TA, Iadecola C (2004). Angiotensin II impairs neurovascular coupling in neocortex through NADPH oxidase-derived radicals. Circ. Res.

[b18] de Laat KF, van den Berg HA, van Norden AG, Gons RA, Olde Rikkert MG, de Leeuw FE (2011). Microbleeds are independently related to gait disturbances in elderly individuals with cerebral small vessel disease. Stroke.

[b19] Lehoux S, Lemarie CA, Esposito B, Lijnen HR, Tedgui A (2004). Pressure-induced matrix metalloproteinase-9 contributes to early hypertensive remodeling. Circulation.

[b20] Lei C, Lin S, Tao W, Hao Z, Liu M, Wu B (2013). Association between cerebral microbleeds and cognitive function: a systematic review. J. Neurol. Neurosurg. Psychiatry.

[b21] Oomen CA, Farkas E, Roman V, van der Beek EM, Luiten PG, Meerlo P (2009). Resveratrol preserves cerebrovascular density and cognitive function in aging mice. Front. Aging Neurosci.

[b22] Pearson KJ, Baur JA, Lewis KN, Peshkin L, Price NL, Labinskyy N, Swindell WR, Kamara D, Minor RK, Perez E, Jamieson HA, Zhang Y, Dunn SR, Sharma K, Pleshko N, Woollett LA, Csiszar A, Ikeno Y, Le Couteur D, Elliott PJ, Becker KG, Navas P, Ingram DK, Wolf NS, Ungvari Z, Sinclair DA, de Cabo R (2008). Resveratrol delays age-related deterioration and mimics transcriptional aspects of dietary restriction without extending life span. Cell Metab.

[b23] Poels MM, Ikram MA, van der Lugt A, Hofman A, Niessen WJ, Krestin GP, Breteler MM, Vernooij MW (2012). Cerebral microbleeds are associated with worse cognitive function: the Rotterdam Scan Study. Neurology.

[b24] Rajan KB, Aggarwal NT, Wilson RS, Everson-Rose SA, Evans DA (2014). Association of cognitive functioning, incident stroke, and mortality in older adults. Stroke.

[b25] Romero JR, Preis SR, Beiser A, DeCarli C, Viswanathan A, Martinez-Ramirez S, Kase CS, Wolf PA, Seshadri S (2014). Risk factors, stroke prevention treatments, and prevalence of cerebral microbleeds in the Framingham Heart Study. Stroke.

[b26] Seo SW, Hwa Lee B, Kim EJ, Chin J, Sun Cho Y, Yoon U, Na DL (2007). Clinical significance of microbleeds in subcortical vascular dementia. Stroke.

[b27] Springo Z, Tarantini S, Toth P, Tucsek Z, Tarantini S, Koller A, Sonntag WE, Csiszar A, Ungvari Z (2015). Aging exacerbates pressure-induced mitochondrial oxidative stress in mouse cerebral arteries. J. Gerontol. Biol. Med. Sci.

[b28] Tarumi T, Ayaz Khan M, Liu J, Tseng BY, Parker R, Riley J, Tinajero C, Zhang R (2014). Cerebral hemodynamics in normal aging: central artery stiffness, wave reflection, and pressure pulsatility. J. Cereb. Blood Flow Metab.

[b29] Toth P, Csiszar A, Tucsek Z, Sosnowska D, Gautam T, Koller A, Schwartzman ML, Sonntag WE, Ungvari Z (2013a). Role of 20-HETE, TRPC channels, and BKCa in dysregulation of pressure-induced Ca2+ signaling and myogenic constriction of cerebral arteries in aged hypertensive mice. Am. J. Physiol. Heart Circ. Physiol.

[b30] Toth P, Tucsek Z, Sosnowska D, Gautam T, Mitschelen M, Tarantini S, Deak F, Koller A, Sonntag WE, Csiszar A, Ungvari Z (2013b). Age-related autoregulatory dysfunction and cerebromicrovascular injury in mice with angiotensin II-induced hypertension. J. Cereb. Blood Flow Metab.

[b31] Toth P, Tarantini S, Tucsek Z, Ashpole NM, Sosnowska D, Gautam T, Ballabh P, Koller A, Sonntag WE, Csiszar A, Ungvari Z (2014). Resveratrol treatment rescues neurovascular coupling in aged mice: role of improved cerebromicrovascular endothelial function and downregulation of NADPH oxidase. Am. J. Physiol. Heart Circ. Physiol.

[b32] Ungvari Z, Csiszar A, Huang A, Kaminski PM, Wolin MS, Koller A (2003). High pressure induces superoxide production in isolated arteries via protein kinase C-dependent activation of NAD(P)H oxidase. Circulation.

[b33] Ungvari Z, Labinskyy N, Mukhopadhyay P, Pinto JT, Bagi Z, Ballabh P, Zhang C, Pacher P, Csiszar A (2009). Resveratrol attenuates mitochondrial oxidative stress in coronary arterial endothelial cells. Am. J. Physiol. Heart Circ. Physiol.

[b34] Ungvari Z, Bagi Z, Feher A, Recchia FA, Sonntag WE, Pearson K, de Cabo R, Csiszar A (2010). Resveratrol confers endothelial protection via activation of the antioxidant transcription factor Nrf2. Am. J. Physiol. Heart Circ. Physiol.

[b35] Wakisaka Y, Miller JD, Chu Y, Baumbach GL, Wilson S, Faraci FM, Sigmund CD, Heistad DD (2008). Oxidative stress through activation of NAD(P)H oxidase in hypertensive mice with spontaneous intracranial hemorrhage. J. Cereb. Blood Flow Metab.

[b36] Wakisaka Y, Chu Y, Miller JD, Rosenberg GA, Heistad DD (2010a). Critical role for copper/zinc-superoxide dismutase in preventing spontaneous intracerebral hemorrhage during acute and chronic hypertension in mice. Stroke.

[b37] Wakisaka Y, Chu Y, Miller JD, Rosenberg GA, Heistad DD (2010b). Spontaneous intracerebral hemorrhage during acute and chronic hypertension in mice. J. Cereb. Blood Flow Metab.

[b38] Wang M, Khazan B, Lakatta EG (2010). Central arterial aging and angiotensin II signaling. Curr Hypertens Rev.

[b39] Wren JD, Garner HR (2004). Shared relationship analysis: ranking set cohesion and commonalities within a literature-derived relationship network. Bioinformatics.

[b40] Yates PA, Sirisriro R, Villemagne VL, Farquharson S, Masters CL, Rowe CC (2011). Cerebral microhemorrhage and brain beta-amyloid in aging and Alzheimer disease. Neurology.

[b41] Zarzuelo MJ, Lopez-Sepulveda R, Sanchez M, Romero M, Gomez-Guzman M, Ungvary Z, Perez-Vizcaino F, Jimenez R, Duarte J (2013). SIRT1 inhibits NADPH oxidase activation and protects endothelial function in the rat aorta: implications for vascular aging. Biochem. Pharmacol.

